# Human Values and Work Engagement: The Mediating Role of Authenticity Among Workers in a Spanish Religious Organization

**DOI:** 10.3389/fpsyg.2020.00076

**Published:** 2020-01-31

**Authors:** Mar Ortiz-Gómez, Antonio Ariza-Montes, Horacio Molina-Sánchez

**Affiliations:** ^1^Financial Economic and Accounting Department, Universidad Loyola Andalucía, Córdoba, Spain; ^2^Department of Management, Universidad Loyola Andalucía, Córdoba, Spain; ^3^Department of Business Administration, Universidad Autónoma de Chile, Santiago, Chile

**Keywords:** human values, authenticity, work engagement, religious organizations, corporate governance, mediating effect, partial least squares

## Abstract

Nowadays religious organizations play a leading role in the third sector, contributing to maintaining the welfare state in a large number of countries in sectors such as health, education or social services, among others. These organizations provide a service to their users, aiming to transmit the predominant values in their mission statement and simultaneously promote both authenticity and work engagement in their employees. Indeed, the purpose of this article is to evaluate the link between human values and work engagement, as well as the mediating role of authenticity in this relationship. To this end, 938 workers of a Catholic religious organization, which constitutes a relatively unexplored context, is employed. To test the research model and hypotheses, this investigation uses PLS (Partial Least Squares). It covers two notable research gaps. First, the results confirm the direct links between human values, authenticity and work engagement within the context of religious organizations. Second, they provide evidence of the mediating role exercised by authenticity in the relationship between human values and work engagement.

## Introduction

Over the last few decades, the study of religious organizations has become increasingly important. These institutions are currently major players within specific areas of the third sector (e.g., education, healthcare and social work), which is essential to maintain a welfare state. In fact, the size of the non-profit entities within the whole of the global economy remains growing and they represent nowadays a significant component of the European economic and social context (Ariza-Montes et al., 2017). These institutions also provide many relevant benefits that are difficult to quantify, such as the local impacts of voluntary work, employment opportunities for some collectives that have been traditionally disadvantaged in terms of labor, and local services (Ayensa, 2011).

Faith-based organizations represent a pluralistic and unique work environment where religious and secular people coexist while working together. The last collective entails a specific degree of heterogeneity that ranges from workers who strongly identify with the institutional objectives to professionals little committed to the organizational goals (Ariza-Montes et al., 2017). The purpose of these institutions lies more in the way they conduct their activity, transmitting their character and charisma, than in the quantity of work they perform. Finding workers who share the predominant values and mission of the organization is a challenge that these entities must face.

Non-profit organizations have been accused of a lack of professionalism in their human capital in comparison with for-profit companies (Dobrai and Farkas, 2010). For instance, these institutions have less capacity to attract and retain talented workers due to their low level of competitiveness in the market. They are usually able to incorporate only those employees who are not highly motivated by monetary compensation. Bacchiega and Borzaga (2003), among others, assume that this issue constitutes a main risk for their long-term survival. All of the above matters reveal the importance of taking action to increase employees’ work engagement, in order to find and maintain authentic workers who share the values of the organization. Moreover, it is important to highlight that spirituality is directly connected to employee engagement (Roof, 2015). Most of the workers in the third and social sectors, especially those from religious institutions, are usually influenced by their ideological backgrounds, such as service vocation, empathy with a series of values, and personal self-actualization (Elson, 2006). This fact makes relevant the necessity of research in human values, where there is a very large investigation gap (Adams, 2016), determining which of them lead workers to be more engaged in these entities.

Religious institutions seem an appropriate context for examining these particular links, because human values are directly related to the personal vocation of their religious employees, and therefore, to authenticity and work engagement in their quotidian job. According to Bickerton et al. (2014), spiritual resources promote the meaning of the jobs and of the perceived capability to fulfill them with success. Consequently, the work engagement of this group, as well as authenticity, must increase through the daily work. This relationship is a main and important point to study, as the wellbeing of the workers also depends on the degree of authenticity that the work environment allows them to show (Ariza-Montes et al., 2017). Therefore, given the described unique features of non-profit religious organizations, it is fundamental to understand how their members feel and act for their long-term survival.

Moreover, although some studies could provide valuable insights to understand how the individual links between personal values, authenticity and work engagement operate, research on non-profit faith-based organizations is virtually non-existent, which emphasizes the significance of this investigation. The personal and professional lives of employees in non-profit religious institutions present a larger overlap between them than in other environments (Ménard and Brunet, 2011; Ariza-Montes et al., 2017). According to these authors, these entities constitute a unique context in which to examine the alignment of human values with professional life.

Based on the above context, this article aims to assess the predictive role of human values on authenticity and work engagement, as well as the mediation exercised by authenticity over the relationship between human values and work engagement. To achieve this purpose, the study is carried out in an extensive international Catholic institution whose social labor is centered on the social work sector and the education sector.

The structure of the paper is as follows. In “Theoretical background and research hypotheses” a revision of the most appropriate literature, as well as the hypotheses and research model, are presented. The “Materials and methods” section details the followed methodology. The “Results” section displays the most significant achieved results. In the “Discussion,” the most relevant empirical outcomes are discussed. The article ends by summarizing the principal conclusions, as well as implications and limitations.

## Theoretical Background and Research Hypotheses

To establish the hypotheses of this research, the framework of this paper reviews in the following paragraphs the theoretical concepts of human values, work engagement and authenticity, as well as the direct and indirect relationships between them.

### Human Values

Values are conceptualized as cognitive representations of universal needs (Schwartz, 1992). Schwartz’s (1992) Theory of Human Values indicates that members of almost all cultures, when they relate to values as guiding principles, implicitly identify ten types of basic human values. Schwartz’s (2006) study indicates that these universal motivational values act together based on a hierarchy of priorities, distinguishing each individual from others and characterizing each person. Values are beliefs that refer to desirable goals and that drive action. These features separate values from related concepts, such as norms or attitudes. Values also guide people in the evaluation of actions, individuals, policies and events. Schwartz (2006) explains that the relative importance of values leads attitudes and behaviors, because human values involve the perceptions of what is good and desirable (such as humility, justice, or success) (Ariza-Montes et al., 2018a).

Schwartz’s(1992, 1994) Theory of Human Values groups ten basic values into four higher-order constructs, constituting two large bipolar dimensions. The first one is *self-transcendence* (universalism and benevolence) versus *self-enhancement* (achievement and power), and the second one is *openness to change* (hedonism, self-direction and stimulation) versus *conservation* (conformity, security and tradition).

On the one hand, self-enhancement or individualism concerns the individual interests of each person and the maximization of his or her potential, while self-transcendence, also known as collectivism, makes reference to a greater concern for the wellbeing of others. On the other hand, the construct of openness to change inspires movement and living new experiences, while conservation motivates individuals to maintain their actual situation in terms of resistance to anything that involves change.

This research considers that values play a main role among employees of religious organizations, where the human values and the personal profile of each individual can condition the interaction between professional and personal roles, ultimately affecting the workers’ experience of authenticity and therefore their work engagement.

### Work Engagement

The positive connection between work and life in different organizational contexts is demonstrated. The benefits of work engagement are not reduced to the work area but also include the personal areas of life, improving the quality of life outside the workplace, as in what healthcare refers to as good social functioning, such as enriching family relationships (Greenhaus and Powell, 2006; Schaufeli et al., 2008; Culbertson et al., 2012; Rodríguez-Muñoz et al., 2014).

Work engagement refers to the positive and continuous emotional affective state of workers. Schaufeli et al. (2002) affirm that it is defined by *absorption*, *dedication* and *vigor*. Absorption means being completely focus on and happily immersed in the job, so that time appears to go quickly. On the other hand, dedication leads to experience a sense of involvement, inspiration, enthusiasm, challenge, meaning and pride. Last, vigor is synonymous with being devoted to work, with energy, pleasure and effort despite difficulties.

The argument of Halbesleben (2010) that engaged workers are more probable to accomplish their tasks than those with a lower degree of work engagement, it is even stronger among employees that have faith in God, as spiritual beliefs reinforce their meaning in the workplace (Park, 2012). This is because religious and spiritual aspects can influence how individuals interpret the occurrences of their daily lives or the way they structure their pursuits, and their general sense of wellbeing and life satisfaction (Emmons, 1999; Lewis and Cruise, 2006). Indeed, a longitudinal study of Christian religious employees (cross-cultural missionaries, clergy, chaplains, and others employed within faith-based institutions) proved that the link with God causes more work engagement than in other collectives (Bickerton et al., 2014).

### Authenticity

Every day, there are increasing numbers of employees who question the meaning of work and how their jobs fit with the other roles in their lives (Hartung, 2009). Scholars from an extensive range of disciplines have drawn attention to the intensifying search of authenticity in developed societies (Liedtka, 2008; Grandey et al., 2012; Van den Bosch and Taris, 2014a). This matter has become increasingly important, as being authentic is beneficial for individuals and collectives, which contributes to generating healthier entities. Many are the psychopathologies that are created in individuals when they are forced to perform behaviors contrary to their nature (de Carvalho et al., 2015).

Authenticity mainly refers to acting in congruence with one’s self, beliefs and core values (Harter, 2002; Ménard and Brunet, 2011; de Carvalho et al., 2015); some humanistic theorists call it respect of one’s needs and values or self-respect (Erikson, 1959; Maslow, 1976). On the other hand, self-determination theories understand authenticity as self-initiated behaviors in line with the inherent basic psychological needs of competence, relatedness, and autonomy (Sheldon and Kasser, 1995; Deci and Ryan, 2000, 1995). According to these latter theories, two dimensions compose authenticity: cognitive and behavioral (Goldman and Kernis, 2002). The cognitive dimension involves the knowledge and appraisal of the self (Deci and Ryan, 2000), while the behavioral dimension refers to one’s true self and acting sincerely in the interactions and relations (Goldman and Kernis, 2002; Kernis and Goldman, 2006). Therefore, authenticity has a long record in philosophy and psychology (Van den Bosch and Taris, 2014a); however, it has received limited attention in scientific research, specifically in the business literature, until very recently, mostly due to there being scarce reliable measures of this concept (Sheldon, 2004; Wood et al., 2008). There is also a particular dimension of spirituality in this term, where one’s authenticity is living in tune with one’s soul or God, not only with one’s belief system or values (Burks and Robbins, 2012).

This research takes Roger’s (1961) definition as a point of reference. This author considers that authenticity is centered on the person. It is an attitude that allows the whole functioning of individuals. Authenticity can be explained by a three-dimensional structure (Wood et al., 2008), which is nowadays the most approved theory among scientific researchers (Ariza-Montes et al., 2017). The three-dimensional model of authenticity developed by Wood et al. (2008) is shaped by *authentic living, accepting external influence* and self-alienation. First, authentic living means being loyal to oneself and behaving by one’s beliefs and values. Second, accepting external influence is understood as complying with the expectations of others; this means in what grade an individual is affected by other people’s thoughts and actions. Finally, self-alienation concerns a state in which a person experiences incongruence between who he or she is and a particular experience; applied to the workplace, self-alienation would be not knowing who one is at work. Therefore, authenticity achieves its maximum level through the combination of a low degree of self-alienation and accepting external influence and a large level of authentic living.

The three-dimensional model of authenticity is very appropriate for studies in the work area (Goldman and Kernis, 2002; Ilies et al., 2005). It is demonstrated that authenticity generates a wide range of positive effects among workers as they find a meaningful job (Ménard and Brunet, 2011; Reich et al., 2013). However, there is a growing need for empirical investigation of authenticity in the workplace (Knoll et al., 2015). Moreover, a large proportion of the current measures consider authenticity to be a stable state instead of relating it to a context (Metin et al., 2016). As far as we know, the concept of authenticity has been studied in different environments, but what human values lead employees to be authentic in their everyday work, and how being authentic contributes to work engagement, among employees of faith-based organizations, have not been examined.

### Direct Relationship Between Human Values and Work Engagement

Human values play an essential role in determining how personality is manifested in behavior (Cropanzano et al., 1992), and an indisputable reality is that human values hold a principal position in institutions with a strong social mission, such as faith-based entities. In addition, as explained before, there is a positive direct relationship between work engagement and spiritual resources, as the link with God generates more work engagement in religious workers than in other groups of people (Bickerton et al., 2014). Among these religious and social employees (both with a pronounced social perspective), collectivism (self-transcendence) prevails over individualism (self-enhancement) (Kim, 2012; Ariza-Montes et al., 2018a). Therefore, self-transcendence (benevolence and universalism) should lead workers of faith-based organizations to be more engaged in their work. Although the relationship between self-transcendent values and work engagement has not been extensively explored, some studies of nurses have investigated this relationship. These research demonstrate that there is a significant positive correlation between self-transcendence (understood by Frankl (1992) as the ability of individuals to discover meaning in their lives by being directed toward something or someone other than themselves, a concept quite similar to Schwartz’s dimension of self-transcendence) and work engagement (Palmer et al., 2010; Tomic and Tomic, 2010; García-Sierra et al., 2015).

Moreover, these groups of religious and social workers are also characterized by features such as tradition, humility, obedience and social order. Furthermore, some of these groups include nuns or other members of religious orders with a high average age (which is usual in Europe) that are used to having stability and order while providing their service to the community (Ariza-Montes et al., 2018a), which places conservation over openness to change in the context of Schwartz’s values. Thus, conservation (understood as tradition, conformity and security by Schwartz’s Theory of Human Values) should motivate work engagement in workers of religious organizations. In fact, some authors (Arciniega and González, 2006) affirm that continuance commitment is an intrinsic value of conservation, as this pole of the dimension comprises values related to security and conformity.

Therefore, these statements lead to the following two hypotheses:

**Hypothesis 1:** Self-transcendence is positively related to work engagement among workers of religious organizations.

**Hypothesis 2:** Conservation is positively related to work engagement among workers of religious organizations.

### Assessing the Mediation Role of Authenticity (Indirect Relationship Between Human Values and Work Engagement)

As explained before, human values hold the main role in determining manifested behavior. In addition, Harter’s (2002) definition of authenticity helps to clarify the relationship between human values and authenticity, as he affirms that the last concept involves that both, feelings and thoughts, must be congruent with actions, leading to authentic behaviors. McCarthy (2015) consider that human beings’ authenticity depends on the consistent pursuit of self-transcendence. He defends that people have a natural capacity for self-transcendence and are universally called to authenticity. McGhee and Grant (2008) consider that spiritual (which entails for him self-transcendence) people seek to live an authentic life. They act spiritually, living selflessly and meaningfully while striving to actualize their ultimate concern, and building authentic relationships with others (Bhaskar, 2013). The studies that examine the relationship between human values and authenticity in daily work are very scarce, and we have not identified any studies conducted in the context of faith-based entities.

Due to this lack of studies, to analyze this relationship, this research focuses on the concept of authentic leadership, as spirituality (understood as self-transcendence, self-sacrifice, and a feeling of meaning and purpose) promotes authentic leadership (Klenke, 2007). First, it is important to note that altruism is an essential aspect of authentic leadership (Gardner et al., 2005). Different studies support that focusing on the needs of others, as the final goal, and the recognition of “compassion,” lead to a positive view of altruistic behavior (Worchel et al., 1988; Batson, 1998). Kanungo and Mendonca (1996) also discuss altruism and its manifested leadership behaviors of cooperation, helping, charity, and motivating others. These researchers argue that altruistic behavior is fundamental for leaders, as they require being receptive to others and showing an interest in the welfare of the institution and its workers, gaining their trust and commitment. These leaders also need to ensure that the vision and the strategy that they are going to implement are in line with the perspectives of others, as well as with their needs and aspirations for collective achievements.

From this point of view, focusing on authentic leadership, Michie and Gooty (2005) discuss the difference between authentic and inauthentic leaders. These authors, together with Howell and Avolio (1992) and Bass and Steidlmeier (1999), point out that only socialized transformational leaders, concerned with the common good, are considered authentic leaders. Leaders with strong integrity are characterized by internal consistency (including feeling emotions that are coherent with self-transcendent values), which leads to acting in line with values that respect the rights and interests of others. Moreover, Michie and Gooty (2005) explain that those honest leaders, who feel respect and compassion for others, act more consistently on these values without emotional conflict, and are therefore more authentic. These statements about the characteristics of authentic leaders align with Schwarz’s self-transcendence construct (benevolence and universalism). Hence, these theories support that self-transcendent values contribute to a work context of high consistency between values and behaviors. Particularly, for this research, the relationship between self-transcendent values and authentic leadership appears to be clear, as most of the managers of the target organization are nuns, or in other words, altruistic leaders who exemplify and demonstrate religious values to others. Therefore, these theories as a whole, building on Schwartz’ values, lead to the following hypothesis:

**Hypothesis 3:** Self-transcendence is positively related to authenticity among workers of religious organizations.

Authenticity, understood as authentic living, accepting external influence and self-alienation (Wood et al., 2008), is more likely to manifest, with greater intensity, among those people who conduct voluntary service. This affirmation is supported by the reason that volunteering is a freely chosen activity (Stebbins, 2004, 2001), and that those volunteers, who feel in an imposed position, role, or identity, contrary to their values, usually choose another voluntary service (Campbell, 2010). This fact leads volunteers to have a free commitment, and therefore, to have a greater degree of authenticity.

Moreover, religious volunteering (Lim and MacGregor, 2012) and participatory activism (Petrova and Tarrow, 2007) are both influenced by personal values. Most people dedicated to volunteering in faith-based organizations or churches place a great deal of importance on God in their lives and pursue traditional values (Ariza-Montes et al., 2018b). Non-secular societies or cultures are more traditional and conservationist and show little tolerance (Inglehart and Baker, 2000), demonstrating an altruistic dedication in volunteering in religious institutions (Choi and DiNitto, 2012; Forbes and Zampelli, 2014; Prouteau and Sardinha, 2015), while secular societies are mostly “modern” and less dedicated to voluntary work (Ariza-Montes et al., 2018b). Then, volunteers in faith-based organizations, who are characterized by conservationist values similar to those of Schwartz, act in an authentic way in their collaborations. However, although this research does not study volunteers, but workers employed by religious organizations, all these studies lead to the hypothesis that conservation (understood as tradition, conformity and security by Schwartz’s Theory of Human Values) motivates authenticity in workers of faith-based institutions. We thus propose the following research hypothesis:

**Hypothesis 4:** Conservation is positively related to authenticity among workers of religious organizations.

There is an increasing need to evaluate the role of authenticity in different areas of life such as work (Ilies et al., 2005). Here, at this point, the question arises as to what extent that work allows employees to act according to their thoughts, beliefs and preferences, is relevant. The research of Sheldon et al. (1997) demonstrates that the low degree of authenticity (across different positions) is related to higher levels of perceived stress, anxiety and depression. Person-Environment (P-E) fit Theory states that stress is a result of the incongruence of the person and his or her environment (Caplan, 1983; Edwards et al., 1998). Misfits between an individual and his or her environment could induce stress and strain, leading to a lower level of wellbeing and work engagement, feeling less comfortable at the work, and losing energy while pretending to be someone else (Van den Bosch and Taris, 2014b). However, workers who feel authentic in their job, being faithful to their values and beliefs, are more intrinsically motivated, being “pulled” toward their work (Van Beek et al., 2012; Emmerich and Rigotti, 2017). In fact, in a study conducted by Ménard and Brunet (2011), managers who perceived that they could be themselves at their jobs tended to find meaning and purpose, as well as satisfaction and emotions, in their occupation. Hence, the perception of having a meaningful job is associated with authenticity.

Moreover, in a study performed by Burks and Robbins (2012), among clinical psychologists, they emphasize the importance of therapists being authentic in their work. These authors notice that the more authentic the therapist could be in a session, the more comfortable the therapists could feel in the conversation, helping them to be more committed to their clients. The study participants admitted that religious beliefs influence these relations, as faith is an intrinsic part of who they are. Being true to one’s inner self is connected to positive outcomes and work engagement (Grandey et al., 2012). Authentic employees should fit their job better than inauthentic workers do and present greater performance (Van den Bosch and Taris, 2014b). This relationship is also extrapolated to the field of leadership. A study performed among army action teams, by Hannah et al. (2011), reveals that team leader authenticity is positively related to team authenticity, which leads to greater team productivity.

Focusing on each of the dimensions of authenticity defined by Wood et al. (2008), to be more authentic, the dimension denominated authentic living should show a high level, while accepting external influence and self-alienation must present a low level. Therefore, a positive relation is supposed to exist between the first dimension and work engagement and a negative relation between the last two dimensions and work engagement. Using a sample of 685 employees, Van den Bosch and Taris (2014b) highlight that authenticity at work accounts for, on average, 11% of the variance of different work outcomes. Self-alienation is the hugest predictor of work engagement, followed by authentic living and accepting external influence. Hence, these authors conclude that employees who feel more authentic in their workplace fit better in it and are more energetic and more engaged in their work. In a more recent investigation, performed with 546 participants, Van den Bosch and Taris (2018) demonstrate that high levels of authenticity at work should be associated with higher levels of work engagement. Moreover, in another research developed by Ariza-Montes et al. (2019) among 208 nuns, whose objective is to study work engagement as a mediator variable between authenticity and subjective wellbeing, they demonstrate that there is a significant direct link between those religious workers who act in accordance with their values and work engagement.

As the validity of the studies performed by Van den Bosch and Taris (2014b, 2018) is limited to just employees working in business and financial services, and those performed by Ariza-Montes et al. (2019) is limited to nuns, this research extrapolates this conclusion to all the workers (religious and secular) of a Catholic non-profit religious organization, due to the importance that this type of institutions currently have. Therefore, this research raises the following hypothesis:

**Hypothesis 5:** Authenticity is positively related to work engagement among workers of religious organizations.

Finally, all these hypotheses lead to the belief that authenticity plays a mediating role between human values and work engagement, as being self-transcendent and conservationist leads not only to a higher level of work engagement, but also to a greater degree of authenticity, which contributes to being more engaged in the workplace. Given these relationships, the following hypotheses are considered:

**Hypothesis 6a:** Authenticity mediates the link between self-transcendent values and work engagement among workers of religious organizations.

**Hypothesis 6b:** Authenticity mediates the link between conservationist values and work engagement among workers of religious organizations.

**FIGURE 1:**
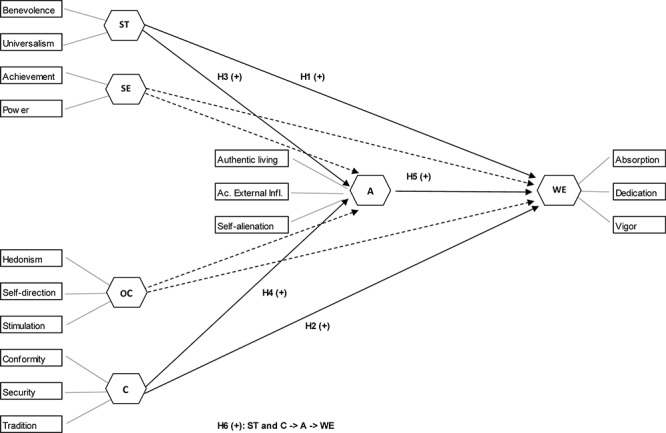
Research model and working hypotheses. A, authenticity; C, conservation; OC, openness to change; SE, self-enhancement; ST, self-transcendence; WE, work engagement.

Figure 1 summarizes the theoretical model and the research hypotheses.

## Methodology

### Sample and Data Collection

To conduct this research, a Google Forms survey was mailed to all members of the target institution, which is a Catholic organization with a wide range of branches throughout Spain. The target organization belongs to a community of apostolic life that was founded in France in the seventeenth century. Subsequently, this company expanded to a large number of countries, such as Spain, Switzerland, Italy, Germany, Portugal, Ireland, Greece or the United States. The institution is currently present in 5 continents (93 countries) with more than 20,000 religious workers. They live and serve in places of social priority: hospitals, homes for orphans, schools, shelters for homeless people or for those who suffer disabilities. The mentioned questionnaire mailed to the target institution was accompanied by an explanation of the goals of this investigation. Before participating in the study, all the individuals gave their informed consent for inclusion. The link to the questionnaire was sent by email to all the respondents, and it was answered on a wide range of devices: computers, smartphones and tablets. All the replies were saved from Google Forms to a spreadsheet in Google Drive. The investigation was performed conforming to the Declaration of Helsinki. The data collection was carried out between April and May 2016. The survey was sent to 1,942 workers, of which 1,014 questionnaires were answered and 938 were valid questionnaires, after rejecting the difference by incomplete parts, resulting in a 48.3% final valid response rate.

Of the 938 respondents, 88.8% are employees, and 11.2% are managers. Moreover, 79.9% are secular, while just 20.1% are religious. Another characteristic of this sample is that most of the workers are women (84.2%; men are just 15.8%), and in terms of sector activity, most of the respondents develop their activity in the education sector (55.2%), and the rest of them belong to the social assistance sector (44.8%), which is formed mainly of social dining rooms, homes for orphans, and residences for elderly people. Other significant demographic data includes that most of the population has completed university studies (70.4%), and the other has finished secondary studies (18.3%) or primary education (11.2%). Finally, the respondents have an average age of 44.9 years.

### Measurements

All the variables in this research are measured through validated questionnaires. To assess human values, as stated in Schwartz’s Theory of Human Values (Schwartz, 1992), the reduced version of PVQ (Portrait Value Questionnaire), composed of 21 items, is employed. This instrument measures 10 fundamental values, classified into four higher-order constructs and two orthogonal axes (self-transcendence – self-enhancement and conservation – openness to change). Each of the items defines a person with whom the surveyed could feel identified or not, using a Likert scale ranging from 1 (in no way the description fits me) to 4 (the description closely resembles me). Some illustrations of items are as follows: “It is important to her/him to understand different people” (universalism – self-transcendence); “It is important to her/him to show abilities and be admired” (achievement – self-enhancement); “It is important to her/him to follow traditions and customs” (tradition – conformity); “It is important to her/him to think new ideas and being creative” (self-direction – openness to change). The validity and reliability of PVQ is demonstrated by Schwartz and Rubel-Lifschitz (2009) in diverse environments, achieving reliability indexes ranging from 0.37 to 0.70. This study achieves good quality criteria as all VIF (Variance Inflation Factor) values are lower than 1.5 (see Table 3).

To measure work engagement, this study employs the Spanish version (produced by Benevides-Pereira et al., 2009) of UWES (Utrecht Work Engagement Scale), which was developed by Schaufeli and Bakker (2003). This scale includes the three dimensions that constitute this variable (absorption, dedication and vigor). Each dimension is measured in the questionnaire by three items, according to a Likert scale ranging from 1 (never) to 5 (always). Then, a larger punctuation represents a higher level of work engagement: absorption (i.e., I feel happy when I am working intensely), dedication (i.e., I am enthusiastic about my job) and vigor (i.e., At my job, I feel strong and vigorous). Different studies (Schaufeli et al., 2006; Demerouti et al., 2015) demonstrate the validity and reliability of this scale. The estimated reliability of this research for the three subscales ranges from 0.723 (absorption) to 0.838 (dedication) (see Table 3).

To assess authenticity at work, Van den Bosch and Taris (2014a) developed the IAM (Individual Authenticity Measure at work), which is an adaptation of the authenticity scale designed by Wood et al. (2008). This questionnaire include the three dimensions discussed in the theoretical framework: authentic living (i.e., “At work, I always stand by what I believe in”), accepting external influence (i.e., “I am strongly influenced in the workplace by the opinions of others”) and self-alienation (i.e., “I don’t feel who I truly am at work”’). Each dimension is composed of 4 items that are ranked applying a Likert scale that ranges from 1 (totally agree) to 5 (totally disagree). Accepting external influence and self-alienation subscales are recoded to be consistent with the subscale for authentic living, in which a higher score represents a greater level of authenticity. Van den Bosch and Taris (2014a) and Metin et al. (2016) demonstrate the scale’s reliability. The reliability estimated in this research ranges from 0.728 (authentic living) to 0.781 (accepting external influence) (see Table 3).

### Data Analysis

This research uses PLS (Partial Least Squares), a variance-based approach of structural equation modeling (SEM) (Roldán and Sánchez-Franco, 2012). This technique was chosen first based on the properties of the constructs involved in the research model. As theoretical contributions (Rigdon, 2012; Henseler et al., 2014) and empirical simulation studies (Becker et al., 2013; Sarstedt et al., 2016) have confirmed, the application of PLS is appropriate to composite measurement models. In this article, the PLS path modeling estimates are consistent (Rigdon, 2016), and there is no bias (Sarstedt et al., 2016). Lastly, this model has been selected for its adaptability to studies carried out in the field of social science research, as the data tend to be non-normally distributed, the measurement scales are frequently poorly developed, theoretical frameworks lack solid development, the focus is mainly on the prediction of the dependent variables, there are enough ordinal and categorical data, and the research model appears to be quite complicated in relation to the type of links defined in the hypotheses (Roldán and Sánchez-Franco, 2012).

Partial least squares permits the evaluation of the reliability and validity of theoretical constructs’ measures, as well as the estimation of the relationships among these constructs (Barroso et al., 2010). This research uses SmartPLS 3.2.8 software, following a two-step approach, to implement the multidimensional superordinate constructs (Chin, 2010). Consequently, using the PLS algorithm, all the items of each dimension are optimally weighted and combined, to build a latent variable score. Later, the first-order factors (dimensions) become the observed indicators of the second-order constructs, which are self-transcendence, self-enhancement, conservation, openness to change, authenticity and work engagement variables (Chin and Gopal, 1995). A construct is a general concept that is estimated either reflective or formative. Hair et al. (2017) explain that if the indicators are highly correlated and interchangeable, they are reflective and estimated in Mode A, and their reliability and validity should be thoroughly examined. Then, their outer loadings, composite reliability, AVE (Average Variance Extracted) and discriminant validity should be examined and reported. However, if the indicators cause the latent variable and are not interchangeable among themselves, they are formative and they will be estimated in Mode B. As such, it is no necessary to report indicator reliability, internal consistency reliability, and discriminant validity. It will be examined the validity, the magnitude and significance of the weights, as well as the multicollinearity of the indicators. In social science research, visualizing the measure as an approximation seems more realistic (Rigdon, 2014), what from a conceptual point of view, favors the use of composite (formative) indicators over causal (reflective) indicators. In this study, self-transcendence, self-enhancement, conservation and openness to change are estimated as formative-formative constructs, authenticity as reflective-formative and work engagement as reflective-reflective (Ringle et al., 2012). This article statistically examines the measurement and structural models (Ringle et al., 2015).

## Results

### Descriptive Statistics

The main descriptive statistics concerning the first-order dimensions are presented in Table 1. As can be observed, the subjects denote a high level of self-transcendence (benevolence: 3.83; universalism: 3.80) and a low level of self-enhancement (achievement: 2.14; power: 1.77), while in other Schwartz’s dimension, conservation shows an elevated mean (tradition: 3.61; security: 3.34; conformity: 3.13) and a medium level of openness to change (self-direction: 3.26; hedonism: 2.81; stimulation: 2.67), being the minimum 1 and the maximum 4 on a Likert scale. Authenticity also shows a remarkable level in all its dimensions (authentic living: 4.24; accepting external influence: 4.08; self-alienation: 3.62; of a minimum level of 1 and maximum of 5). Last, all dimensions of work engagement denote an elevated mean (dedication: 4.55; vigor: 4.31; absorption: 4.26; of a minimum level of 1 and maximum of 5). Table 1 also reveals that most of the correlations between dimensions are statically significant and consistent with the suggested models (Modes A and B).

**TABLE 1:** Descriptive statistics and inter-correlations for the study dimensions.

**Variable**	**Mean**	***SD***	**1**	**2**	**3**	**4**	**5**	**6**	**7**	**8**	**9**	**10**	**11**	**12**	**13**	**14**	**15**	**16**
1 Benevolence	3.83	0.39	1															
2 Universalism	3.80	0.37	–0.033	1														
3 Achievement	2.14	0.89	–0.013	0.292**	1													
4 Power	1.77	0.65	0.197**	0.076*	0.161**	1												
5 Conformity	3.13	0.77	0.180**	0.119**	0.220**	0.003	1											
6 Security	3.34	0.70	0.216**	0.156**	0.134**	0.310**	–0.025	1										
7 Tradition	3.61	0.55	–0.006	0.603**	0.269**	0.138**	0.185**	0.083*	1									
8 Hedonism	2.81	0.86	0.239**	0.040	0.103**	0.289**	0.036	0.401**	0.051	1								
9 Self-direction	3.26	0.64	−0.161**	0.186**	0.087**	−0.090**	−0.089**	−0.100**	0.053	–0.058	1							
10 Stimulation	2.67	0.79	−0.223**	0.092**	0.029	−0.074*	−0.171**	−0.072*	–0.020	–0.052	0.473**	1						
11 Authentic living	4.24	0.65	0.427**	0.052	0.028	0.186**	0.139**	0.275**	0.105**	0.333**	−0.174**	−0.249**	1					
12 Ac. external influence	4.08	1.00	0.012	0.201**	0.365**	0.013	0.290**	0.058	0.230**	0.110**	–0.018	–0.048	0.029	1				
13 Self-alienation	3.62	1.02	–0.023	0.418**	0.280**	0.175**	0.065*	0.079*	0.337**	0.084**	0.188**	0.115**	0.050	0.122**	1			
14 Absorption	4.26	0.70	0.027	0.340**	0.514**	0.194**	0.163**	0.102**	0.251**	0.194**	0.039	–0.017	0.038	0.336**	0.250**	1		
15 Dedication	4.55	0.65	0.235**	0.083*	0.228**	0.166**	0.359**	0.081*	0.192**	0.224**	−0.126**	−0.155**	0.295**	0.291**	0.126**	0.263**	1	
16 Vigor	4.31	0.70	–0.062	0.749**	0.295**	0.064*	0.158**	0.133**	0.575**	0.028	0.189**	0.157**	–0.017	0.242**	0.387**	0.286**	0.076*	1

**p < 0.01; *p < 0.05.

### Common Method Bias

Before assessing a PLS model, a statistical technique is employed to identify a potential CMB (Common Method Bias) situation. This approach consists of a full collinearity test based on VIFs (Variance Inflation Factors) to assess both vertical and lateral collinearity. A VIF achieving a value higher than 3.3 indicated pathological collinearity. This indication warned that a model could be contaminated by CMB (Kock and Lynn, 2012; Kock, 2015). As displayed in Table 2, the present model is free of CMB, as it attains a maximum VIF of 1.380.

**TABLE 2:** Full collinearity VIFs.

	**A**	**WE**
A		1.151
C	1.240	1.242
OC	1.067	1.080
SE	1.010	1.013
ST	1.279	1.380

**A, authenticity; C, conservation; OC, openness to change; SE, self-enhancement; ST, self-transcendence; WE, work engagement.**

### PLS Models

To assess PLS results, we follow a two stages approach: first, testing the reliability and validity of both measurement models and, second, evaluating the significance of the paths between the constructs of the structural model. Lastly, we assess the predictive validity of the research model.

#### Measurement Models

Both measurement models, measurement model 1 (for first-order dimensions) in Table 3, and measurement model 2 (for second-order constructs) in Table 4, show acceptable results. Both measurement models satisfy the requirements of item reliability, as the loadings of those first-order dimensions and second-order constructs estimated on Mode A are generally higher than 0.707 (Tables 3, 4; Carmines and Zeller, 1979). Just two of the outer loadings of the indicators are slightly below this critical level (Table 3). However, we decide to maintain them to keep the content validity of the scale (Hair et al., 2011). They also satisfy the requirements of construct reliability, as the Cronbach’s alpha, Jöreskog’s rho (rho_A) and composite reliability (CR) are higher than 0.7 (Tables 3, 4; Nunnally and Bernstein, 1967). Last, all first-order dimensions and second-order constructs reach convergent validity since the AVE is over the 0.5 critical level (Tables 3, 4; Fornell and Larcker, 1981). Finally, Tables 3, 4 also show that based on the Fornell-Larcker criterion (Henseler et al., 2015), diagonal elements (Tables 3, 4) are the square root of the variance shared between the constructs and their measures (AVE). Therefore, those estimated on Mode A satisfy the discriminant validity requirements, as diagonal elements are higher than off-diagonal elements, with off-diagonal items representing the correlations among the constructs.

**TABLE 3:** Measurement model 1 and reliability and validity.

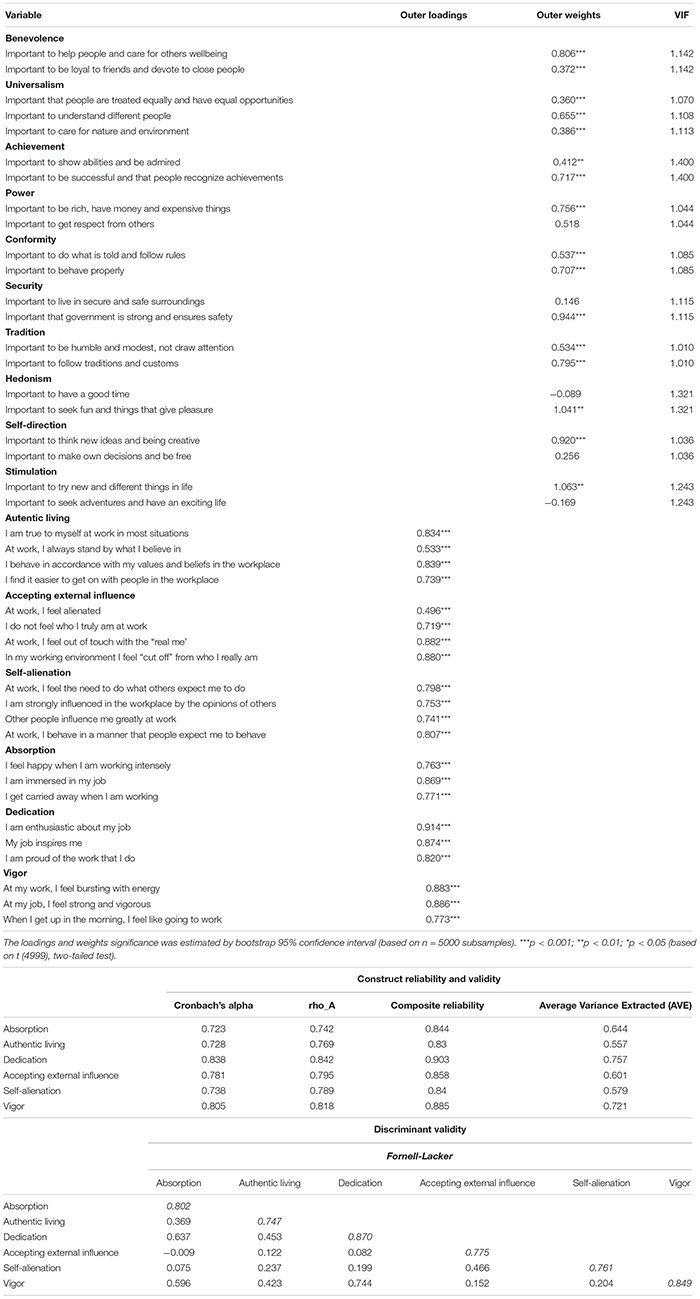

**TABLE 4:** Measurement model 2 and reliability and validity.

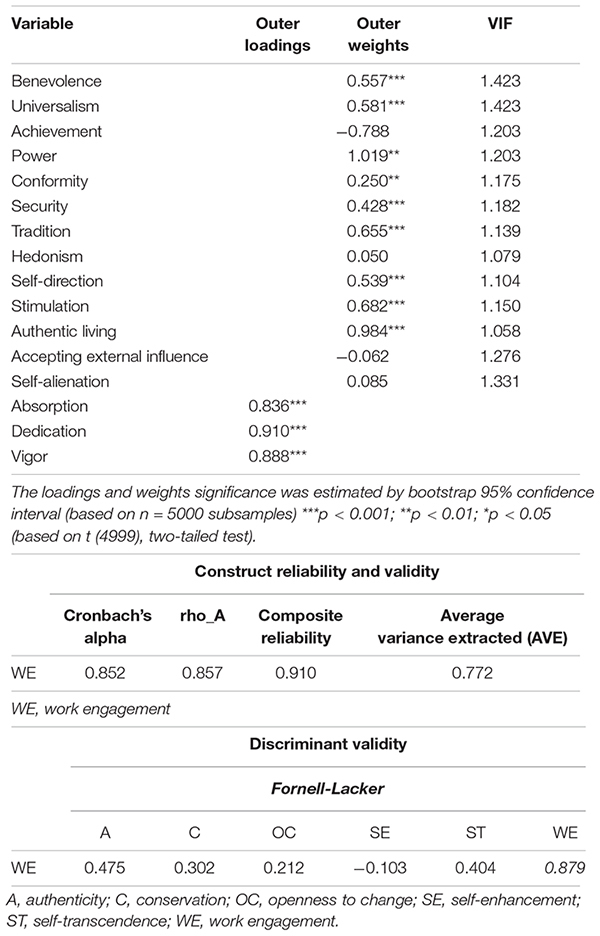

**A, authenticity; C, conservation; OC, openness to change; SE, self-enhancement; ST, self-transcendence; WE, work engagement.**

Concerning those first-order dimensions and second-order constructs estimated on Mode B, the examination starts by testing the potential multicollinearity between the items (Roldán and Sánchez-Franco, 2012). Petter et al. (2007) affirm that a VIF value greater than 3.3 is a signal of high multicollinearity. Nevertheless, Ringle et al. (2015) defend that multicollinearity should be a concern only if VIF values are over the 5 critical level. In this case, the maximum VIF statistic for first-order dimensions and second-order constructs is 1.423, below both thresholds, so multicollinearity is not a concern. Finally, this investigation examines the magnitude and significance of the weights (Tables 3, 4). Weights offer data concerning how each item contributes to the respective dimensions and constructs (Chin, 1998), allowing to place the indicators according to their contribution. A measure is relevant for a composite construct when the significance level is at least 0.05 (Roldán and Sánchez-Franco, 2012). Hence, in both models estimated in Mode B, most of the measures are significant (Tables 3, 4). We decide to maintain all of them to keep the content validity of the scale (Hair et al., 2011).

#### Structural Model

In accordance with Hair et al.’s (2014), this research applies a bootstrapping technique (5,000 re-samples) to produce the standard errors, *t*-statistics, *p*-values and 95% BCCIs (Bias-Corrected Confidence Intervals). They permit the evaluation of the statistical significance for the hypothesized relationships (both direct and indirect). Table 5 displays the principal parameters obtained to assess the structural model. The main criterion for measuring the explained variance of the endogenous constructs is the coefficient of determination (*R*^2^). Our results show that the structural model presents acceptable predictive relevance for the endogenous construct work engagement (*R*^2^ = 0.319). The mediating variable authenticity offers a lower coefficient of determination (*R*^2^ = 0.134), which is because it is a construct that contributes to explaining the variance of work engagement and is in part explained by the constructs of human values, but most of its variance is not explicated by the constructs (Table 5).

**TABLE 5:** Structural model.

***R^2^ WE = 0.319 R^2^ A = 0.134* relationship**	**Path coefficient**	***T*-statistics**	***P*-values**	**2.5%**	**97.5%**	**Significance**
***Direct Effects***						
**ST - > A**	**0.306**	**5.002**	0.000***	**0.183**	**0.421**	**Sig.**
**ST - > WE**	**0.196**	**3.862**	0.000***	**0.095**	**0.294**	**Sig.**
SE - > A	−0.056	1.049	0.294	−0.154	0.069	No Sig.
SE - > WE	−0.052	1.260	0.208	−0.116	0.047	No Sig.
C - > A	0.019	0.459	0.647	−0.072	0.095	No Sig.
**C - > WE**	**0.140**	**4.363**	0.000***	**0.075**	**0.201**	**Sig.**
**OC - > A**	**0.090**	**2.524**	0.012*	**0.009**	**0.152**	**Sig.**
**OC - > WE**	**0.081**	**2.418**	0.016*	**0.013**	**0.142**	**Sig.**
**A - > WE**	**0.367**	**8.721**	0.000***	**0.283**	**0.447**	**Sig.**
***Indirect Effects***						
**ST - > A - > WE**	**0.112**	**4.387**	0.000***	**0.067**	**0.167**	**Sig.**
SE - > A - > WE	−0.021	1.022	0.307	−0.061	0.023	No Sig.
C - > A - > WE	0.007	0.460	0.646	−0.026	0.035	No Sig.
**OC - > A - > WE**	**0.033**	**2.507**	0.012*	**0.004**	**0.057**	**Sig.**

A, authenticity; C, conservation; OC, openness to change; SE, self-enhancement; ST, self-transcendence; WE, work engagement. Bootstrapping 95% confidence intervals bias corrected (based on n = 5000 subsamples). ***p < 0.001; **p < 0.01; *p < 0.05 [based on t (4999), two-tailed test]. Relevant relationships in bold.

As shown in Table 5, the structural model confirms the direct and positive relationships between the dimensions of both self-transcendence (H1) (path coefficient: 0.196^∗∗∗^; *t*-value: 3.862) and conservation (H2) (path coefficient: 0.140^∗∗∗^; *t*-value: 4.363) of Schwartz’s human values and work engagement, confirming that there is no direct relationship between the opposite dimension of self-transcendence, which is self-enhancement, and work engagement. Hence, these results lead to the conclusion that there is empirical evidence to support H1 and H2. However, surprisingly, they show that there is also a direct relationship between the opposite dimension of conservation according to Schwartz, which is openness to change, and work engagement (path coefficient: 0.081^∗^; *t*-value: 2.418), although this direct relationship is less intense than the first one (conservation–work engagement), which is supported by the literature.

The structural model (Table 5) also supports the direct and positive relationship between self-transcendence and authenticity (H3) (path coefficient: 0.306^∗∗∗^; *t*-value: 5.002), rejecting the relationship between its opposite dimension, self-enhancement and authenticity. Nevertheless, the results do not support a direct relationship between conservation and authenticity (H4) (path coefficient: 0.019; *t*-value: 0.459), supporting the direct relation of its opposite dimension, openness to change–authenticity (path coefficient: 0.090^∗^; *t*-value: 2.524). Hence, these results contribute to the conclusion that there is empirical evidence to sustain H3, as well as the opposite dimension of H4 (openness to change–authenticity). This structural model also describes a significant positive direct effect between authenticity and work engagement (H5) (path coefficient: 0.367^∗∗∗^; *t*-value: 8.721), which means that there is empirical evidence to sustain H5.

This article also conducts a mediation analysis. In PLS a step-wise approach is not necessary, as it is able to test mediating effects in a single model at once (Nitzl et al., 2016). The steps proposed by Zhao et al. (2010), and later supported by others authors such as Nitzl et al. (2016) and Hair et al. (2017), for the mediator analysis procedure are the following: first, determining the significance of the indirect effect; second, determining the type of effect or of mediation. Then, this model proves that there is an indirect positive and significant relationship between self-transcendence and work engagement (H6a) (path coefficient: 0.112^∗∗∗^; *t*-value: 4.387), partially mediated by authenticity, as the direct effect self-transcendence-work engagement is also significant and positive (rejecting a significant indirect effect of authenticity on the self-enhancement-work engagement link). There is also empirical evidence to sustain the indirect positive and significant relationship between openness to change and work engagement (path coefficient: 0.033^∗^; *t*-value: 2.507). This link is partially mediated by authenticity, as the direct effect openness to change-work engagement is also significant and positive (rejecting H6b, since the model shows that there is not a significant indirect relationship between conservation and work engagement, and then, no mediation). Thus, the results lead authenticity to be a mediating variable between human values and work engagement, being a complementary partial mediation (Zhao et al., 2010; Nitzl et al., 2016; Hair et al., 2017).

#### Assessment of the Predictive Validity Using Holdout Samples

This research also aims to develop a prediction model. Explanation and prediction follow two different aims that could be combined in an investigation (Shmueli, 2010; Dolce et al., 2017). A model’s predictive ability refers to the capability of producing accurate predictions of further observations, independent of their temporal or cross-sectional nature (Shmueli and Koppius, 2011). Predictive validity explains that a given group of measures, of a specific construct, can predict a certain outcome variable (Straub et al., 2004). Hence, this investigation evaluates the predictive ability of the suggested research model, through the use of cross-validation with holdout samples (Evermann and Tate, 2016), employing the PLS predict algorithm (Shmueli et al., 2016) available in the SmartPLS software version 3.2.8 (Ringle et al., 2015). To assess whether the research model entails predictive ability, this study checks the *Q*^2^ value. Positive *Q*^2^ values indicate that the prediction error of PLS results is smaller than the prediction error of just utilizing the mean values. In this way, the RMSE (Root Mean Squared Error) and the MAE (Mean Absolute Error) are the statics used to predict error. Therefore, positive *Q*^2^ values indicate that the proposed research model presents appropriate predictive ability. Consequently, due to the findings explained above, the research model has enough evidence to confirm its predictive validity (out-of-sample prediction), to forecast values for new cases of the dimensions of authentic living, dedication, vigor and absorption, as well as for all the indicators (Table 6). Therefore, the proposed research model of this article obtains additional support from this predictive validity.

**TABLE 6:** Partial least squares prediction assessment.

**Construct Prediction Summary**		**Dimension Prediction Summary**	

	***Q*^2^**		***Q*^2^**
A	–0.26	Autentic living	0.108
WE	–0.06	Accepting external influence	–0.005
		Self-alienation	–0.005
		Dedication	0.153
		Vigor	0.132
		Absorption	0.137

	**Indicator Prediction Summary**								
	
	**PLS**			**LM**			**PLS-LM**		
			
	**RMSE**	**MAE**	**Q^2^_predict**	**RMSE**	**MAE**	**Q^2^_predict**	**RMSE**	**MAE**	**Q^2^_predict**

I am true to myself at work in most situations	0.724	0.581	16.502	0.078	0.730	0.577	0.646	−0.149	15.925
At work, I always stand by what I believe in	1.071	0.879	36.690	0.019	1.067	0.860	1.052	−0.188	35.830
I behave in accordance with my values and beliefs in the workplace	0.763	0.571	17.906	0.065	0.769	0.571	0.698	−0.198	17.335
I find it easier to get on with people in the workplace	0.889	0.663	23.429	0.063	0.900	0.664	0.826	−0.237	22.765
At work, I feel alienated	1.417	1.223	57.083	0.013	1.410	1.211	1.404	−0.187	55.872
I do not feel who I truly am at work	1.351	1.101	50.862	0.008	1.356	1.098	1.343	−0.255	49.764
At work, I feel out of touch with the “real me’	1.185	0.907	38.927	0.016	1.193	0.909	1.169	−0.286	38.018
In my working environment I feel “cut off” from who I really am	1.128	0.851	35.979	0.032	1.134	0.848	1.096	−0.283	35.131
At work, I feel the need to do what others expect me to do	1.363	1.154	56.269	0.047	1.365	1.154	1.316	−0.211	55.115
I am strongly influenced in the workplace by the opinions of others	1.102	0.878	33.997	0.030	1.108	0.879	1.072	−0.23	33.118
Other people influence me greatly at work	1.140	0.939	36.325	0.031	1.146	0.943	1.109	−0.207	35.382
At work, I behave in a manner that people expect me to behave	1.320	1.107	52.607	0.072	1.330	1.111	1.248	−0.223	51.496
I feel happy when I am working intensely	0.778	0.618	18.185	0.067	0.777	0.608	0.711	−0.159	17.577
I am immersed in my job	0.698	0.560	15.211	0.107	0.700	0.559	0.591	−0.140	14.652
I get carried away when I am working	1.015	0.797	30.009	0.062	1.018	0.798	0.953	−0.221	29.211
I am enthusiastic about my job	0.721	0.567	15.806	0.126	0.719	0.560	0.595	−0.152	15.246
My job inspires me	0.776	0.600	18.056	0.089	0.769	0.592	0.687	−0.169	17.464
I am proud of the work that I do	0.600	0.425	11.812	0.120	0.609	0.426	0.480	−0.184	11.386
At my work, I feel bursting with energy	0.776	0.634	17.883	0.091	0.778	0.633	0.685	−0.144	17.250
At my job, I feel strong and vigorous	0.703	0.588	15.620	0.114	0.709	0.592	0.589	−0.121	15.028
When I get up in the morning, I feel like going to work	0.886	0.702	22.378	0.069	0.874	0.681	0.817	−0.172	21.697

## Discussion

The study of religious organizations is increasingly important. These entities have become main players in particular activities of the services sector (i.e., social services, education and healthcare), and their contributions are essential to maintain the welfare state. In fact, the number of non-profit entities continues to expand within the global economy, and, nowadays, they play a leading role in the European economic and social framework (Ariza-Montes et al., 2017). These organizations do not consider the maximization of their economic value as an end. In contrast, their inspiring principles lie in other sets of priorities that are not of economic nature, such as aligning people with the identity values of the organization.

The current study analyses the role of human values as a significant predictor of work engagement and examines the mediating function of authenticity in this relationship. These links have rarely been addressed, much less in the unexplored context of faith-based entities. To achieve this goal, a Catholic religious organization with a strong presence in Spain is studied, in which approximately 1,000 workers of the educational and social sector are analyzed. In addition, an integral model of the mentioned relationship between human values, authenticity and work engagement is designed, in which both direct and indirect links are proposed. To this end, a model of structural equations is applied to verify the hypotheses raised in this study.

As will be verified below, the achieved results provide very valuable evidence to understand the functioning of religious organizations in critical aspects for their long-term survival, such as the work engagement of their employees.

First, the main claim of this research is that certain human values contribute positively to increasing work engagement among employees of religious organizations. Self-transcendent, and interestingly enough, both of the poles of the dimension conservation versus openness to change (although the latter less intensely), may be related to greater work engagement in these entities. In this line, other studies have confirmed that values predict a series of actions and that these relationships seem to be causal (Verplanken and Holland, 2002; Sagiv et al., 2011). Then, the obtained results are consistent with previous investigations that studied the relationship between self-transcendence and work engagement among nurses (Palmer et al., 2010; Tomic and Tomic, 2010; García-Sierra et al., 2015). These findings suggest that given the social work carried out by religious organizations, altruism is an essential value for achieving the mobilization and selfless commitment of its employees, which will necessarily result in a better quality of service.

Second, the results affirming that conservationist workers may be engaged in faith-based institutions are also in line with investigations explaining that these groups are characterized by values such as tradition, obedience, social order and humility (Ariza-Montes et al., 2018a). In these entities, there is a positive direct relationship between spiritual resources and work engagement (Bickerton et al., 2014). Moreover, some authors such as Arciniega and González (2006) defend conservation is a predictor of continuance commitment. They explain that this commitment or perceived cost of leaving the company is an intrinsic value of conservation, as some groups feel a moral obligation to remain within an organization. In the study entity, there are groups of nuns or other workers, who have spent most of their work lives in this organization, that are used to provide their service with order and stability.

Third, the results suggesting that hedonism, stimulation and self-direction are also positively related to work engagement are consistent with previous investigations among workers from non-religious for-profit entities (Schaufeli et al., 2001; Langelaan, 2007). These authors find that engaged employees feel energetic and in control, are intensely involved in demanding and challenging tasks, and are flexible and open to change, adapting quickly to modifications of their environment. This last relationship may explain why engaged workers keep looking for new tasks in their jobs (Sonnentag, 2003), moving from them when they no longer feel challenged (Schaufeli et al., 2001). However, our findings probably offer the first empirical evidence to validate the relationships between self-transcendent, conservationist and open to change values and work engagement among workers of religious organizations.

Fourth, this article considers authenticity as an end in itself for faith-based entities. Then, it proposes that self-transcendent and conservationist values exert a positive impact on authenticity in employees of religious organizations. This approach is not fully validated since the results confirm that while self-transcendent workers are more authentic, the hypothesis about conservation is not supported. Surprisingly, it is suggested that those who are open to change are the ones who exemplify authenticity. The obtained findings about self-transcendent employees are consistent with the results of authors studying the personality of authentic leaders (Howell and Avolio, 1992; Bass and Steidlmeier, 1999; Michie and Gooty, 2005); however, the results achieved in the present article could offer the first empirical evidence to validate the relationship between self-transcendence and authenticity in workers in religious entities. On the other hand, our conclusions about open to change employees are not in line with previous studies performed with volunteers, who are characterized by conservationist values (Inglehart and Baker, 2000) and act in an authentic way in their volunteerism (Campbell, 2010). Moreover, as far as we know, the relationship between both poles of the last dimension (conservation-openness to change) and authenticity has not been studied among personnel of religious organizations. The importance of authenticity for workers is in concordance with other investigations that affirm that young employees currently choose jobs that match their own personal values (Sortheix et al., 2015; Jonkmans et al., 2016). They want to feel that they can express who they are at their jobs, without being judged negatively or missing development and promotion opportunities. Employees who feel more inauthentic are more likely to behave unethically, resulting in workplace misconduct, such as dishonest financial or social behavior (Ebrahimi et al., 2019). The predominant values of this group are stimulation, self-direction and hedonism (Crumpacker and Crumpacker, 2007), which constitute the openness to change dimension. These studies about young workers could explain the unexpected results of positive relations between openness to change and authenticity.

Fifth, we tested the hypothesis of whether authenticity has a positive relationship with work engagement among employees of religious organizations. The developed partial least squares analysis confirms that those people who can act in accordance with their ideas and beliefs in the workplace present higher levels of vigor, dedication and absorption. These conclusions are in line with prior studies that probe that authenticity in the workplace increases work engagement (Grandey et al., 2012; Van den Bosch and Taris, 2014b, 2018; Ariza-Montes et al., 2019); however, our results could be placed among the first studies of the personnel (secular and religious) of social faith-based entities. Due to the strong demands associated with many of the jobs that are carried out in the social sector (in which workers deal with terminally ill people, battered women or children with serious disabilities, among others), it is likely that the level of authenticity and work engagement, in these employees, is greater than those of workers in different sectors of activity. Therefore, the confirmation of this hypothesis acquires greater relevance in the analyzed context, allowing those workers who live in a more authentic way with their activity to be more engaged and therefore transmit their values while providing the service at the same time as those of the organization.

Finally, this research examines the mediating function of authenticity in the relationship between human values (hypothesizing self-transcendence and conservation) and work engagement in workers of religious entities, which, to the best of our knowledge, has not been addressed before. Authenticity constitutes a fundamental piece in this relationship since being comfortable and acting in a way consistent with one’s beliefs and personal values can be a determining factor in the development of feelings of belonging to different groups, perhaps especially so in faith-based entities (Ariza-Montes et al., 2017). Moreover, the capability of being authentic in the workplace is conditioned by organizational goals (Freeman and Auster, 2011). Hence, the main contribution of this research lies in demonstrating that the probability of being more engaged in the organization should increase among those self-transcendent and open to change members who can act authentically, according to their values and beliefs at work. However, although for those individuals who present self-transcendent and open to change values, a strategy of authenticity at work would increase their work engagement, the results show that this could not be an appropriate option for conservationist workers. Then, these conclusions convert authenticity into an instrument of the organization to help to increase the engagement of those workers who hold specific human values. In addition, it is noteworthy that low levels of self-enhancement values do not contribute to more work engagement or more authenticity. Here arises a very controversial issue and conclusion, since it is a matter of maximization of self-transcendent values but not minimization of self-enhancement values. These results contribute to the governance of religious institutions to identify what types of values should be sought after when selecting potential employees or what kinds of attitudes work with actual employees. Low engagement in the organization is an unsatisfactory situation that affects not only the company but also the individual (Schnell et al., 2013). In fact, the average age of religious workers is getting higher, and most of the time, they are the people who are leading these entities. In the very near future, given the lack of religious vocations, lay members will have to assume the direction of much of the social work that is currently carried out by religious entities. This makes it quite important to identify those lay employees who act in accordance with their beliefs, share the institution’s values and are engaged in their jobs to continue to provide the services of the organization while transmitting its values.

Employees play a fundamental role in the corporate image that an organization transmits to society. This statement acquires even more importance in service entities, given the close relationship that exists between the service provider and the service user. This statement is even stronger in social services organizations, whether they are religious or not. The present investigation confirms that the human values that guide the character of the employees of the analyzed entity are benevolence and universalism, which are positively related to a higher level of authenticity and work engagement. Then, the self-transcendence, authenticity and work engagement of employees should be projected outward (to the general public, to users, to public administration, etc.), contributing to improving the reputational corporate image of the institution in its closest environment.

This article obtains notable implications when examining the most intense values and feelings of workers. The relevant implications include both theoretical (generating healthier work environments in which workers can act in accordance with their values and beliefs and are more engaged in their work, which is a very useful contribution to the governance of these organizations) and practical results (identifying within religious institutions those human values that increase the level of authenticity and work engagement of their workers, and designing preventive policies that increase these levels). Any progress in the direction of human values and emotions of individuals in the workplace improves the functioning of institutions and promotes services to enrich the society, what is the final goal of these institutions. Additionally, this study adds the opportunity to improve the lives of workers of faith-based entities, with strategies that allow them to be more authentic according to their thoughts and beliefs, while simultaneously increasing their work engagement. These circumstances could advise the implementation of training activities oriented to improve the levels of authenticity of the employees of these institutions.

## Conclusion

The conclusions derived from this research are consistent with the idiosyncrasies that characterize religious institutions. The faith-based entity analyzed in this article exhibits two main aspects by being a religious organization (whose principal purpose is transmitting its institutional values) and a service institution. First, this religious circumstance implies that its objective is not only to have engaged employees but also to have employees who live their work in an authentic way (Canda, 1989). The fact that authenticity is one of the main goals of this type of institution is what probably makes the research model works, something that could not occur in a profit and non-religious company. The personality of the individuals working in them is also in line with the results, as they are usually people who care about others and appreciate places that allow them to act in accordance with their ideas and beliefs (Ariza-Montes et al., 2017), or in other words, people with high levels of self-transcendent values and authenticity. Second, the faith-based organization is not the only differentiating factor; so too is the sector to which it belongs. Usually, the activities that are developed in the social service sector, such as in residences for the elderly or educational entities, are vocational (Elson, 2006). This means that values such as societal contribution, social justice, work-life balance and supportive management practices prevail in their workers (Winter and Jackson, 2014). Social environments demand social skills, reward helpful behavior, provide opportunities for the appearance of compassion or sympathy, and encourage the presentation of cooperative and charitable values. Hence, employees working in the social field show a personality characterized by interpersonal skills, prefer working with people to working with things, and value social service and caring or educating others (Don Gottfredson and Duffy, 2008). These characteristics of the social sector highlight the relevance of being engaged at work, as generally, these jobs are personally demanding. This range of demands means that, in some cases, people working in this sector prefer an entity that shares their values and allows them to develop as a person, although it implies, for instance, a lower salary, than another one with more advantageous economic conditions that do not enable them to be authentic. Authenticity is very valued by employees (Ménard and Brunet, 2011; Reich et al., 2013), and most of the entities try to be a model in this concept, becoming an objective itself and a way to achieve work engagement.

Hence, this study covers a large investigation gap in the relatively unexplored context of religious organizations, demonstrating the fundamental role that human values play as predictors of authenticity and work engagement, and that authenticity mediates the relationship between human values and work engagement. Two valuable conclusions are obtained from this research. First, the more self-transcendent and conservationist (or open to change, although less intensely) the workers of religious organizations are, the more engaged they may be in their work. Second, in this relationship, there is a mediating role exercised by authenticity (which is an end in itself for faith-based institutions), which makes this variable a key feature to work on. Following this last strategy, those workers who are self-transcendent and open to change could be more engaged in their work and within the organization.

## Limitations and Future Research Lines

In spite of the contributions, both theoretical and practical, this research is not without some methodological limitations. First, the information was obtained through self-reports, which could cause a response bias, which, according to de Carvalho et al. (2015), could be improved with objective measures. Second, although the results of this research could be extrapolated to other faith-based organizations and other companies in the third sector, they are based on the Catholic institution where the research was conducted, and even though it has an international perspective, this institution is placed in the particular geographic area of Spain. Third, the research model implies two chains that flow in the first case from a predictor variable (human values) to a mediator variable (authenticity) to an outcome variable (work engagement), and in the second case directly from the predictor variable to the outcome variable. Nevertheless, such propositions should not be rigorously assessed based on the cross-sectional data available for this research. Longitudinal data would help to address the possible existence of causal relations between these variables. Finally, another limitation is that while PLS is appropriate for investigations developed within the social sciences, it also has some caveats that should be taken into account in the analysis of the results (e.g., McIntosh et al., 2014).

This manuscript also counts with some other additional limitations. The high value placed on tradition, conformity and security, within the target religious organization, could likely be because the sample of this study is mainly composed of women workers who are into middle and deep age. Some investigations, such as Adams (2016), say that women are more conservationist than men are and that they reinforce these values as years go by. Then, some future lines of investigation could incorporate age and gender as moderator variables of the studied relationships.

Although this research is developed in the context of a religious organization, some of the obtained evidence could be useful for for-profit companies. These companies are increasingly looking for new management models that go beyond economic incentives and allow workers to find meaning in their work, thereby achieving engaged workers. In addition, workers are increasingly searching for companies that allow them to act according to their values and beliefs. Future research lines could prove that this model is also valid in for-profit entities.

## Data Availability Statement

The datasets generated for this study are available on request to the corresponding author.

## Ethics Statement

Ethical review and approval was not required for the study on human participants in accordance with the local legislation and institutional requirements. The patients/participants provided their written informed consent to participate in this study.

## Author Contributions

All authors listed have made a substantial, direct and intellectual contribution to the work, and approved it for publication.

## Conflict of Interest

The authors declare that the research was conducted in the absence of any commercial or financial relationships that could be construed as a potential conflict of interest.
